# NDVI Changes Show Warming Increases the Length of the Green Season at Tundra Communities in Northern Alaska: A Fine-Scale Analysis

**DOI:** 10.3389/fpls.2020.01174

**Published:** 2020-07-31

**Authors:** Jeremy L. May, Robert D. Hollister, Katlyn R. Betway, Jacob A. Harris, Craig E. Tweedie, Jeffrey M. Welker, William A. Gould, Steven F. Oberbauer

**Affiliations:** ^1^ Department of Biological Sciences, Florida International University, Miami, FL, United States; ^2^ Department of Biological Sciences, Grand Valley State University, Allendale, MI, United States; ^3^ Department of Biological Sciences, University of Texas at El Paso, El Paso, TX, United States; ^4^ Ecology and Genetics Research Unit, University of Oulu, Finland & UArctic, Oulu, Finland; ^5^ Department of Biological Sciences, University of Alaska Anchorage, Anchorage, AK, United States; ^6^ USDA Forest Service International Institute of Tropical Forestry, Rio Piedras, Puerto Rico

**Keywords:** Arctic shrubs, Normalized Difference Vegetation Index, climate change, seasonality, phenology, International Tundra Experiment, experimental warming

## Abstract

A warming Arctic has been associated with increases in aboveground plant biomass, specifically shrubs, and changes in vegetation cover. However, the magnitude and direction of changes in NDVI have not been consistent across different tundra types. Here we examine the responsiveness of fine-scale NDVI values to experimental warming at eight sites in northern Alaska, United States. Warming in our eight sites ranged in duration from 2‑23 seasons. Dry, wet and moist tundra communities were monitored for canopy surface temperatures and NDVI in ambient and experimentally-warmed plots at near-daily frequencies during the summer of 2017 to assess the impact of the warming treatment on the magnitude and timing of greening. Experimental warming increased canopy-level surface temperatures across all sites (+0.47 to +3.14˚C), with the strongest warming effect occurring during June and July and for the southernmost sites. Green-up was accelerated by warming at six sites, and autumn senescence was delayed at five sites. Warming increased the magnitude of peak NDVI values at five sites, decreased it at one site, and at two sites it did not change. Warming resulted in earlier peak NDVI at three sites and no significant change in the other sites. Shrub and graminoid cover was positively correlated with the magnitude of peak NDVI (r=0.37 to 0.60) while cryptogam influence was mixed. The magnitude and timing of peak NDVI showed considerable variability across sites. Warming extended the duration of the summer green season at most sites due to accelerated greening in the spring and delayed senescence in the autumn. We show that in a warmer Arctic (as simulated by our experiment) the timing and total period of carbon gain may change. Our results suggest these changes are dependent on community composition and abundance of specific growth forms and therefore will likely impact net primary productivity and trophic interactions.

## Introduction

Warming in the Arctic has been accelerating in recent decades, and the Arctic is experiencing more pronounced temperature increases compared with lower latitudes (ACIA, 2005; Serreze et al., 2009; Stocker et al., 2013). Accompanying increased temperatures are decreases in snow cover and increases in growing season length (Callaghan et al., 2011; Liston and Hiemstra, 2011). Adaptations (e.g. slow growth) to the inherently harsh growing conditions of the Arctic have caused plants to be sensitive to small variations in temperature that can be reflected in interannual variation in growth and in phenology (Callaghan et al., 1999). Measurable changes in Arctic plant growth and community dominance in response to warming have been well documented (Arft et al., 1999; Oberbauer et al., 2013; Khorsand Rosa et al., 2015; Bjorkman et al., 2015; Elmendorf et al., 2015; Bjorkman et al., 2018). However, these responses have not been uniform across the Arctic and are likely associated with local climate conditions and ecohydrology including snow and its role in growing season length and tundra plant ecophysiology (Elmendorf et al., 2012a; May et al., 2017; Prevéy et al., 2017; Jespersen et al., 2018).

In response to changes in environmental conditions over recent decades, an expansion northward and within local landscapes of shrubs and graminoids has been documented in the Arctic (Tape et al., 2006; Myers-Smith et al., 2011; Elmendorf et al., 2012b; Tape et al., 2012; Myers-Smith et al., 2015). Documenting and understanding shifts in community dominance and ecosystem function are important to accurately predict the trajectories and impact of climate change on Arctic systems. This includes how these changes are manifested in the seasonality of growth (Welker et al., 1997; Pearson et al., 2013) and the degree to which the abundance of vegetation may be expressed in spectral properties that can be measured at plot and landscape scales (Reidel et al., 2005; Raynolds et al., 2008; Gamon et al., 2013).

The immense area and remoteness of the region have made remote-sensing tools indispensable in monitoring the effects of climate change in the region (Kerr and Ostrovsky, 2003; Stow et al., 2007; Walker et al., 2012; Ju and Masek, 2016). Despite their vast area, Arctic plant communities are spatially heterogeneous at very small scales (m), due in part to differences in hydrology as result of small variation in topography (cm, Billings and Bliss, 1959; Evans et al., 1989; Walker et al., 1989; Ostendorf and Reynolds, 1998, Lara et al., 2018; Shaefer and Messier, 1995). Normalized Difference Vegetation Index (NDVI), developed by Kriegler et al. (1969), has proven to be a valuable and widely-used tool in monitoring productivity and community dominance changes across the Arctic (Reidel et al., 2005; Raynolds et al., 2008; Berner et al., 2018). Studies using remotely-sensed NDVI data have demonstrated a trend toward a greening Arctic over recent decades (Jia et al., 2003; Stow et al., 2007; Bhatt et al., 2013; Keenan and Riley, 2018), although some regions have shown browning (Phoenix and Bjerke, 2016; Zhang et al., 2017; Myers-Smith et al., 2020). In spite of the time and resource saving advantages of remotely-sensed monitoring tools at large spatial scales, the high spatial heterogeneity of Arctic tundra complicates our ability to understand and predict how specific communities respond to varying environmental conditions and across latitudinal gradients (Verbyla, 2008; Gamon et al., 2013; Guay et al., 2014; Healey et al., 2014; Reichle et al., 2018). Linking vegetation sampling with measurements of NDVI at small spatial scales should be a useful approach to improve understanding of the complexity of observed large-scale NDVI changes over time. Previous studies have focused on NDVI changes over time with regards to ambient conditions over large spatial scales (Blok et al., 2011; Gamon et al., 2013; Pattison et al., 2015; Ju and Masek, 2016) or with warming focused on a specific community type (Boelman et al., 2005). The approach that we are implementing here, however, could also be very useful when applied to ecosystem responses to experimental warming across a variety of community types and latitudes at fine temporal and spatial scales.

Species responses to experimental warming have been examined in a variety of community types across the tundra biome using traditional manual measurement approaches (Chapin and Shaver, 1985; Wookey et al., 1993; Parsons et al., 1994; Arft et al., 1999). The International Tundra Experiment (ITEX) was established in 1990 as a circumpolar network of researchers to quantify plant, community, and ecosystem process changes in the Arctic in response to experimental warming (Webber and Walker, 1991; Molau and Molgaard, 1996; Henry and Molau, 1997; Welker et al., 1997). The use of open-top chambers (OTCs) in standardized, plot-scale passive warming in tundra plant communities across polar and alpine regions has been an extremely powerful approach to understand tundra responses to both experimental and background climate warming (Arft et al., 1999; Elmendorf et al., 2012a; Elmendorf et al., 2012b; Oberbauer et al., 2013; Leffler et al., 2016; Jespersen et al., 2018). The ITEX network has shown that experimental warming causes increased growth and early phenological development in the Arctic (Wookey et al., 1993; Arft et al., 1999; Barrett et al., 2015; Bjorkman et al., 2015; Prevéy et al., 2017) as well as shifts in community dominance (Elmendorf et al., 2012a; Elmendorf et al., 2012b; Hollister et al., 2015), although the magnitude of responses varies greatly across sites. Studies of community diversity and dominance have shown that warming temperatures favor tall-statured species, such as shrubs and graminoids, and decrease cover of some cryptogams (Elmendorf et al., 2012b; Hollister et al., 2015). However, how these vegetation changes correspond to changes in timing and magnitude of NDVI at fine-scales are uncertain (Huemmrich et al., 2013; Zesati, 2017), especially as a result of experimental warming.

Here we present the results from a study of canopy-level surface temperature and NDVI across the growing season at eight ITEX sites in northern Alaska for which we had detailed plot-level measurements of plant community composition. The canopy-level surface temperature measurements allowed testing of the effectiveness of the warming treatment. The use of an LED-illuminated NDVI sensor provided high precision data allowing documentation of daily changes in NDVI independent of sky conditions. The overarching goals of this study were to: 1) test the effect of experimental warming on spring green-up and green season length, 2) identify when NDVI is near or at peak values in different tundra communities, and 3) investigate how plant community composition influences the magnitude and timing of peak NDVI values. We predicted that experimental warming increases peak NDVI values, a proxy for peak productivity, and extend the green season length at all sites. We also predicted that communities dominated by shrubs and graminoids would exhibit greater increases in peak NDVI values with warming due to increased aboveground vegetation, compared to communities with greater cryptogam dominance.

## Methods and Materials

### Site Description

**Figure 1:**
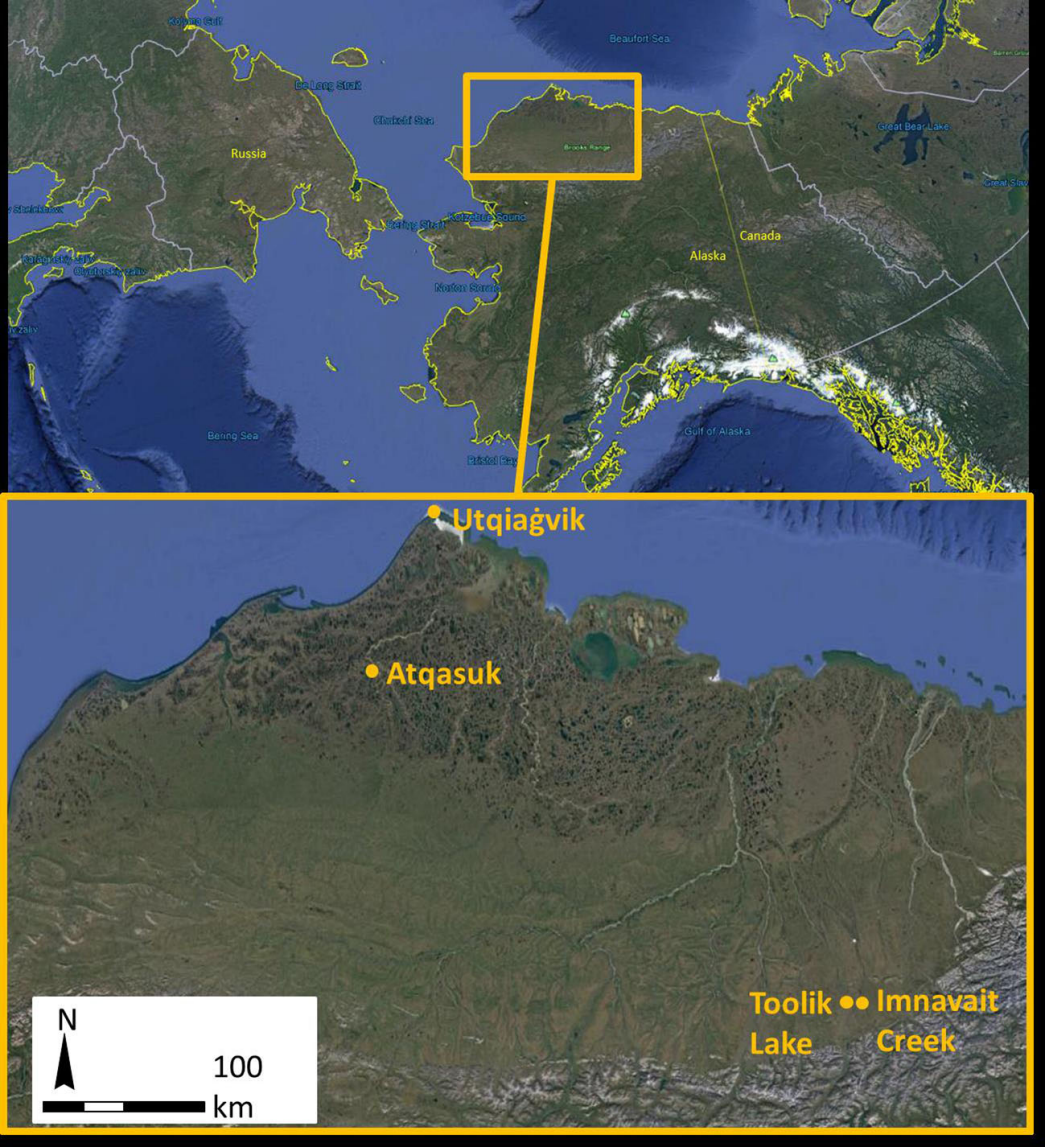
Locations of ITEX sites on the North Slope, Alaska, United States.

Eight study sites, a paired wet/moist and dry site, were arrayed across four regions of the North Slope of Alaska, USA. The regions provide a latitudinal gradient spanning Low to High Arctic from the northern foothills of the Brooks Range to the northern Alaska coast along the Arctic Ocean (Welker et al., 2005, **Figure 1**). Toolik Lake and Imnavait Creek are both inland, foothill tundra, Atqasuk is inland, positioned at the transition between foothill and coastal plain tundra, and Utqiaġvik is coastal plain tundra. Study sites at Utqiaġvik (formerly Barrow), Atqasuk, Imnavait Creek, and Toolik Lake, Alaska each consisted of a paired dry site and a wet or moist community site. Sites at Utqiaġvik, Atqasuk, and Toolik Lake were established between 1994 and 1997 (20‑23 seasons of warming), and Imnavait Creek sites were established in 2016 (2 seasons of warming; Hollister et al., 2005; Walker et al., 2006). The Utqiaġvik dry site (71˚18’48.46”N, 156˚35’5.67”W) is located on an old beach ridge above a drained thaw-lake bed and is dominated by short-statured shrubs, graminoids, and lichens (**Table 1**). The Utqiaġvik wet site (71˚18’40.98”N, 156˚35’53.70”W) is located on a frequently inundated slope between a beach ridge and drained thaw-lake basin and is dominated by graminoids and bryophytes. The Atqasuk dry site (70˚27’13.81”N, 157˚24’25.37”W) is located on a well-drained ridge and is dominated by short-statured shrubs and lichens. The Atqasuk wet site (70˚27’11.33”N, 157˚23’59.61”W) is located in a frequently inundated meadow and is dominated by deciduous shrubs, graminoids, and bryophytes. The Imnavait Creek dry site (68˚36’58.37”N, 149˚18’21.49”W) is located on a well-drained slope and is dominated by short-statured shrubs and lichens. The Imnavait Creek wet site (68°36’ 56.25” N, 149˚18’ 21.17”W) is located on a slope within the head of a water track feature and is dominated by shrubs, graminoids, and bryophytes. The Toolik Lake dry site (68°37’19.04”N, 149°35’53.71”W) is located on a well-drained ridge and is dominated by short-statured shrubs and lichen. The Toolik Lake moist site (68°37’12.33”N, 149°36’12.04”W) is located on a partially drained, gradual slope with acidic soils and dominated by graminoids (mainly *Eriophorum vaginatum* tussocks), shrubs, and bryophytes. The Imnavait Creek and Toolik Lake sites are at very similar latitudes, but Imnavait Creek is at higher elevations by almost 200 m (927 vs 736 m a.s.l.) and therefore is generally cooler. Warmed and control plots have similar species present in the communities, however, differ in abundances (Hollister et al., 2015). The number of sampled control and warmed plots, as well as dominant species varied among sites (**Table 1**). Plots were not paired and were randomly arranged across the landscape of each community type.

**Table 1:** Number of plots for each treatment (warmed or control) within each study site and dominant species present.

	Utqiaġvik	Atqasuk	Imnavait Creek	Toolik Lake
	Dry	Dry	Dry	Dry
**Number of Plots**	19	19	8	10
**Year Established**	1994	1996	2016	1995
**Latitude**	71^o^18’48.46" N	70^o^27’13.81" N	68^o^36’58.37" N	68^o^37’19.04" N
**Longitude**	156^o^35’5.67" W	157^o^24’25.37" W	149^o^18’21.49" W	149^o^35’53.71" W
**Landscape Orientation**	Drained thaw lake beach ridge	Frequently inundated meadow	Well-drained slope	Well-drained ridge
**Dominant Species**	*Luzula confusa*	*Ledum palustre*	*Arctostophylos alpina*	*Arctostophylos alpina*
	*Luzula arctica*	*Vaccinium vitis-idaea*	*Vaccinium vitis-idaea*	*Vaccinium vitis-idaea*
	*Salix rotundifolia*	*Cassiope tetragona*	*Cassiope tetragona*	*Cassiope tetragona*
	*Vaccinium vitis-idaea*	*Carex bigelowii*	*Betula nana*	*Betula nana*
	*Pedicularis kaneii*	*Hierchloe alpina*	*Hierochloe alpina*	*Carex bigelowii*
** **	Lichen	Lichen	Lichen	Lichen
	Wet	Wet	Wet	Moist
**Number of Plots**	19	19	8	10
**Year Established**	1995	1996	2016	1995
**Latitude**	71^o^18’40.98" N	70^o^27’11.33" N	68^o^36’56.25" N	68^o^37’12.33" N
**Longitude**	156^o^35’53.70" W	157^o^23’59.61" W	149^o^18’21.17" W	149^o^36’12.04" W
**Landscape Orientation**	Frequently inundate beach slope	Well-drained ridge	Head of a water-tract feature	Gradual acidic slope
**Dominant Species**	*Carex aquatilis-stans*	*Carex aquatilis*	*Eriophorum vaginatum*	*Eriophorum vaginatum*
	*Dupontia fisherii*	*Eriophorum angustifolium*	*Salix pulchra*	*Salix pulchra*
	*Eriophorum angustifolium*	*Eriophorum russeolum*	*Betula nana*	*Betula nana*
	*Eriophorum russeolum*	*Salix pulchra*	*Ledum palustre*	*Ledum palustre*
	Pleurocarpus moss	*Salix polaris*	*Carex bigelowii*	*Carex bigelowii*
	Sphagnum spp.	Pleurocarpus moss	*Cassiope tetragona*	*Vaccinium vitis-idaea*
		Sphagnum spp.	Pleurocarpus moss	Pleurocarpus moss
			Sphagnum spp.	Sphagnum spp.

### Treatment and Measurement

Control and warmed plots were ~1m^2^ in size across all sites. Warming was achieved using hexagonal open-top chambers (OTCs) that were installed shortly after snowmelt and removed at the end of the growing season each year (Marion et al., 1997). The OTCs were constructed of Sun-Lite^®^ HPTM fiberglass (Kalwall Corporation, Manchester, New Hampshire, USA) and installed according to the guidelines outlined in the ITEX manual (Molau and Mølgaard, 1996). Open-top chambers have been shown to warm surface air temperatures by an average of 0.6 to 2.2°C over the summer which is analogous to climate change projections (Marion et al., 1997; Hollister et al., 2006). A non-destructive point frame method was performed for vegetation community sampling according to Walker (1996). The point frame method consists of a 75 cm x 75 cm 100 point frame with measurement points every 7 cm that was leveled above the canopy. Permanent markers were used to ensure the frame was installed in the same position and orientation at each sampling. At each point a graduated ruler was lowered to the vegetation where species, live/dead status, and height were measured at the top (canopy level) and bottom (understory) contact point. All Utqiaġvik and Atqasuk sites were sampled in 2017, Imnavait Creek sites were sampled in 2016, and Toolik Lake sites were sampled in 2014. All sites were included for analysis despite the asynchrony of vegetation sampling because samplings are conducted independently for the larger research project of vegetation monitoring. All point frame samplings took place during peak growing season (mid- to late July; for details see Hollister et al., 2015 and Hobbie et al., 2017).

During the growing season (June‑August) of 2017, canopy surface temperatures and NDVI values were recorded at each plot near solar noon (1:00‑3:00 PM AKST) on an almost-daily basis (every 1‑2 days). Canopy surface temperature was measured using Fluke 62 Max^®^ infrared thermometers (Fluke Corporation, Everett, Washington, United States). As a result of a logistic issue, canopy surface temperature measurements for the month of June at the Utqiaġvik and Atqasuk sites were replaced with air temperatures recorded at a height of ~10cm using thermocouples contained in six plate Gill radiation shields at 1300 h (Campbell Scientific, Inc., Logan, Utah, USA; Robert Hollister, unpublished data). NDVI was measured using a handheld, LED-illuminated NDVI reader (GreenSeeker^®^,Trimble Navigation Ltd., Sunnyvale, CA, USA) at a height of approximately 50cm held perpendicular to the ground. Care was taken to ensure that the footprint of both GreenSeeker^®^ and the infrared thermometer were similar on each plot using the GreenSeeker^®^ LEDs and the laser on the infrared thermometer. The LED illumination of the GreenSeeker^®^ is brighter than ambient light and thus minimizes measurement differences resulting from inconsistent sky conditions and uneven solar illumination of the surface. The GreenSeeker^®^ uses the normalized difference between near-infrared (R_774_) and red (R_656_) light wavelength reflectance to calculate NDVI [NDVI = (R_774_ - R_656_)/(R_774_ + R_656_)].

### Data Analysis

Daily mean canopy level surface temperatures for control and warmed plots were aggregated by month (June, July, and August) and combined into a full-season value in order to better compare whole month values between sites. Temperatures in each treatment were compared for each month and full season using repeated measure analysis of variance (RANOVA). The maximum seasonal NDVI value of each site was used to characterize peak NDVI, length of green season, and spring green-up. Peak NDVI was defined as the 1st day that NDVI values were above 95% of the maximum seasonal NDVI value for the season. We chose 95% of the maximum because there was a clear plateau during the summer around which NDVI values increased in spring or declined in fall. Daily changes in NDVI values have been shown to correspond to temperatures experienced within the prior 3 days (May et al., 2017), as a result, the values fluctuate a small amount throughout the season. The length of the green season was defined as the time period when NDVI values remained above 95% of the maximum seasonal NDVI value until senescence caused NDVI values to decrease below 95%. Spring green-up was defined as the time period when NDVI values were increasing between 80% and 95% of the maximum seasonal NDVI value; we recognize a longer window would be ideal, but we were logistically constrained by the need to compare across all sites and the length of the field season. Warming effects on the magnitude and the timing (day of year) of peak NDVI were compared for each site using analysis of variance (ANOVA); the length of the green season and the daily change in NDVI (calculated by average increase in NDVI value) during spring green-up were also compared for each site using ANOVA. The relationship between plant growth form percent cover and the timing and magnitude of peak NDVI was tested using a series of Pearson Correlations. All statistical tests were performed using the R statistical environment (R Core Team, 2018, Vienna, Austria).

## Results

### Warming Effects on Surface Temperature

Full-season average canopy surface temperatures varied along the latitudinal gradient of study sites, with the effectiveness of the OTC warming greater at the southernmost sites compared to the northernmost sites (**Table 2**). Warming effectiveness was variable across sites and among months during the summer. Effectiveness of OTC warming in the dry sites was lower (+0.47 to +2.65°C) compared to the wet and moist sites (+1.37 to +3.14°C). Warming treatment significantly increased June temperatures at most sites with the exception of the dry sites at Utqiaġvik (+0.01°C p=0.966), Atqasuk (+0.75°C p=0.504), and Toolik Lake (+.2.22°C p=0.163). In July, OTC warming increased canopy surface temperatures at all sites in the northernmost regions of Utqiaġvik (dry +0.83°C p=0.012 and wet +2.51°C p<0.001) and Atqasuk (dry +1.49°C p=0.016 and wet +1.83°C p<0.001). In July, the only site within the southernmost regions that showed significant increases in canopy surface temperature with OTC warming was Imnavait Creek dry site (+2.69°C p=0.047). In August, only the Utqiaġvik wet site (+0.96°C p=0.015) showed significant increases in canopy surface temperature with OTC warming.

**Table 2:** Average canopy surface temperature for control and experimentally-(OTC) warmed plots at all sites during the months of June, July, August, and all months combined.

Community	June			July			August			Full Season		
	Control	Warmed	p-value	Control	Warmed	p-value	Control	Warmed	p-value	Control	Warmed	p-value
**Utqiaġvik**												
Dry	*0.74*	*0.0*1	0.966	**4.69**	**0.83**	**0.012**	4.13	0.55	0.131	3.19	0.47	0.136
Wet	***-0.05***	***0.63***	***0.007***	**4.38**	**2.51**	**<0.001**	**3.72**	**0.96**	**0.015**	**2.72**	**1.37**	**<0.001**
**Atqasuk**												
Dry	*5.88*	*0.75*	*0.504*	**11.08**	**1.49**	**0.016**	7.65	0.07	0.934	8.14	0.77	0.153
Wet	***3.03***	***1.39***	***0.034***	**6.56**	**1.83**	**<0.001**	5.62	1.2	0.068	**5.07**	**1.47**	**<0.001**
**Imnavait Creek**												
Dry	**14.63**	**3.06**	**<0.001**	**20.51**	**2.69**	**0.047**	14.62	2.22	0.285	**16.61**	**2.65**	**0.023**
Wet	**13.71**	**3.79**	**0.007**	21.58	1.85	0.354	15.01	3.15	0.101	**16.76**	**2.93**	**0.031**
**Toolik Lake**												
Dry	14.16	2.22	0.163	20.18	1.94	0.136	14.13	1.67	0.375	16.33	1.94	0.081
Moist	**14.63**	**1.83**	**0.005**	20.67	2.28	0.368	15.28	3.23	0.097	**16.73**	**3.14**	**0.041**

Warmed temperatures are presented as the average increase in temperature over the control plot values. Italicized values indicate shielded air temperatures collected at 10cm height in Utqiaġvik and Atqasuk in June (used a surrogate for missing surface temperatures). Bold indicates a statistically significant difference between control and warmed plot values (based on ANOVA, p < 0.05).

### OTC Warming Effects on NDVI

**Figure 2:**
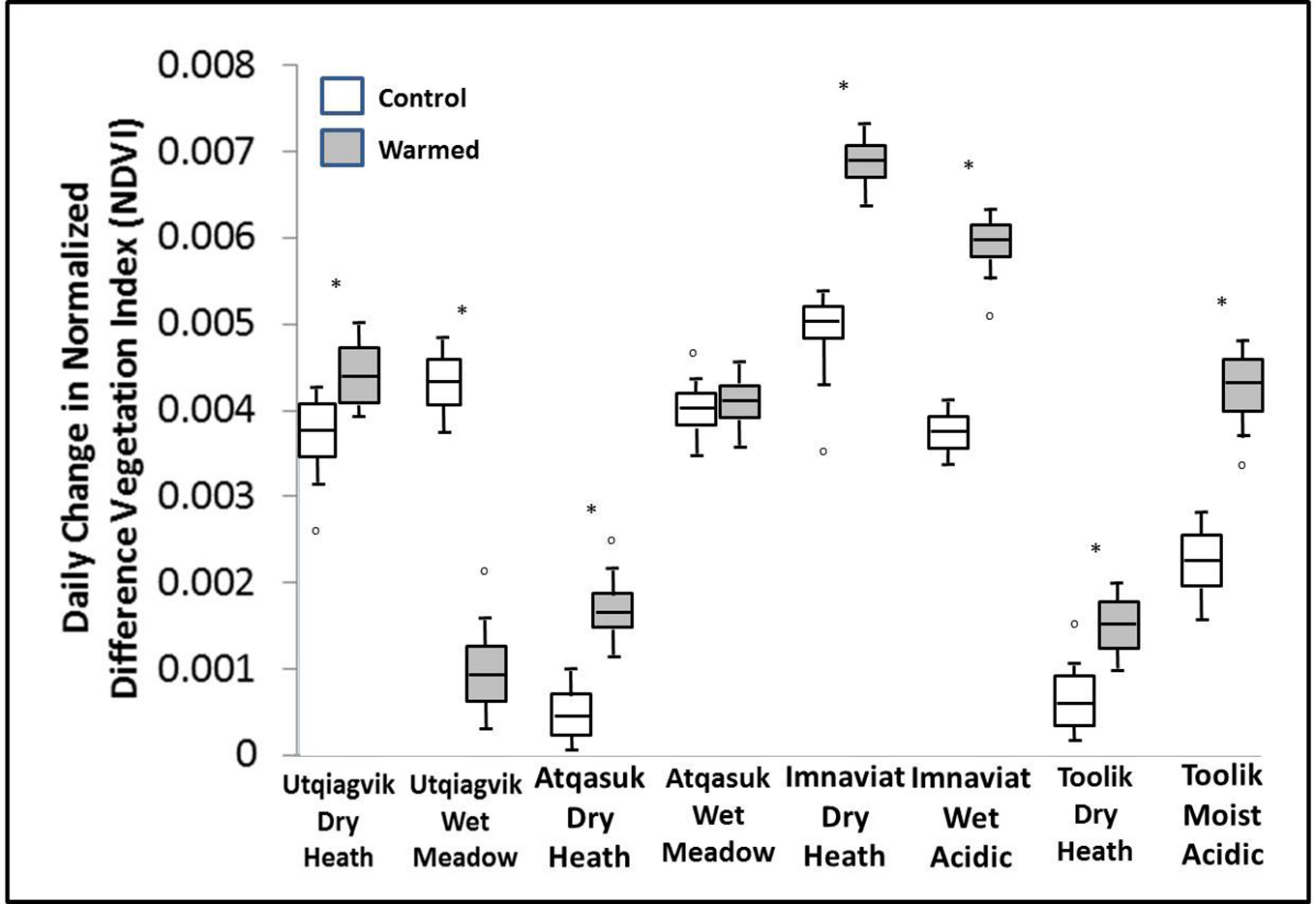
Average daily change in NDVI during spring green-up in control (open) and warmed (filled) plots at the eight study sites. A * denotes a statistically significant difference between treatments at the site (based on ANOVA, p < 0.05).

**Figure 3:**
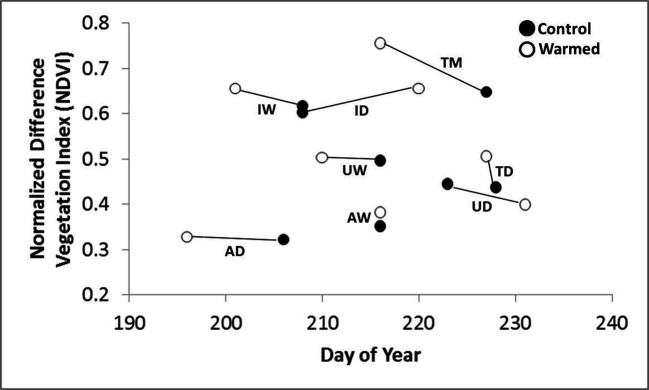
The average peak NDVI magnitude and day of occurrence for control (filled circle) and warmed (open circle) plots at the eight study sites (UD- Utqiaġvik dry, UW- Utqiaġvik wet, AD-Atqasuk dry, AW-Atqasuk wet, ID-Imnavait Creek dry, IW-Imnavait Creek wet, TD-Toolik Lake dry, TM-Toolik Lake moist).

Warming treatment significantly increased the daily change in NDVI during spring green-up at all sites except Atqasuk dry and Imnavait wet (**Figure 2**). The sites that showed the largest increase were Imnavait Creek dry (+0.003 NDVI day^-1^ p=0.008) and Toolik Lake moist (+0.006 NDVI day^-1^ p<0.001). Warming treatment increased the magnitude of peak NDVI values at most sites; however, OTC warming had mixed effects on the timing of peak NDVI (**Figure 3**). Earlier peak NDVI occurred at the Atqasuk dry (-7 days p=0.012), Imnavait Creek dry (-9 days p=0.007), and Toolik Lake moist (-22 days p<0.001) sites with no significant change at the remaining sites (average differences of -2 days to +3 days).

Warming treatment increased the magnitude of peak NDVI the most at the Toolik Lake dry (+0.065 NDVI p<0.001) and moist (+0.103 NDVI p<0.001) sites, followed by the dry (+0.050 NDVI p=0.007) and wet (+0.036 NDVI p=0.029) sites at Imnavait Creek (**Figure 3**). Warming treatment at the Atqasuk sites showed mixed results with the peak NDVI at the wet site increasing (+0.029 NDVI p=0.048) while the dry site remaining unchanged (+0.006 NDVI p=0.561). The Utqiaġvik wet site peak NDVI value did not change with OTC warming (+0.005 NDVI p=0.582), and the dry site was the only site that showed a decrease in peak NDVI with OTC warming (-0.043 NDVI p=0.012).

**Figure 4:**
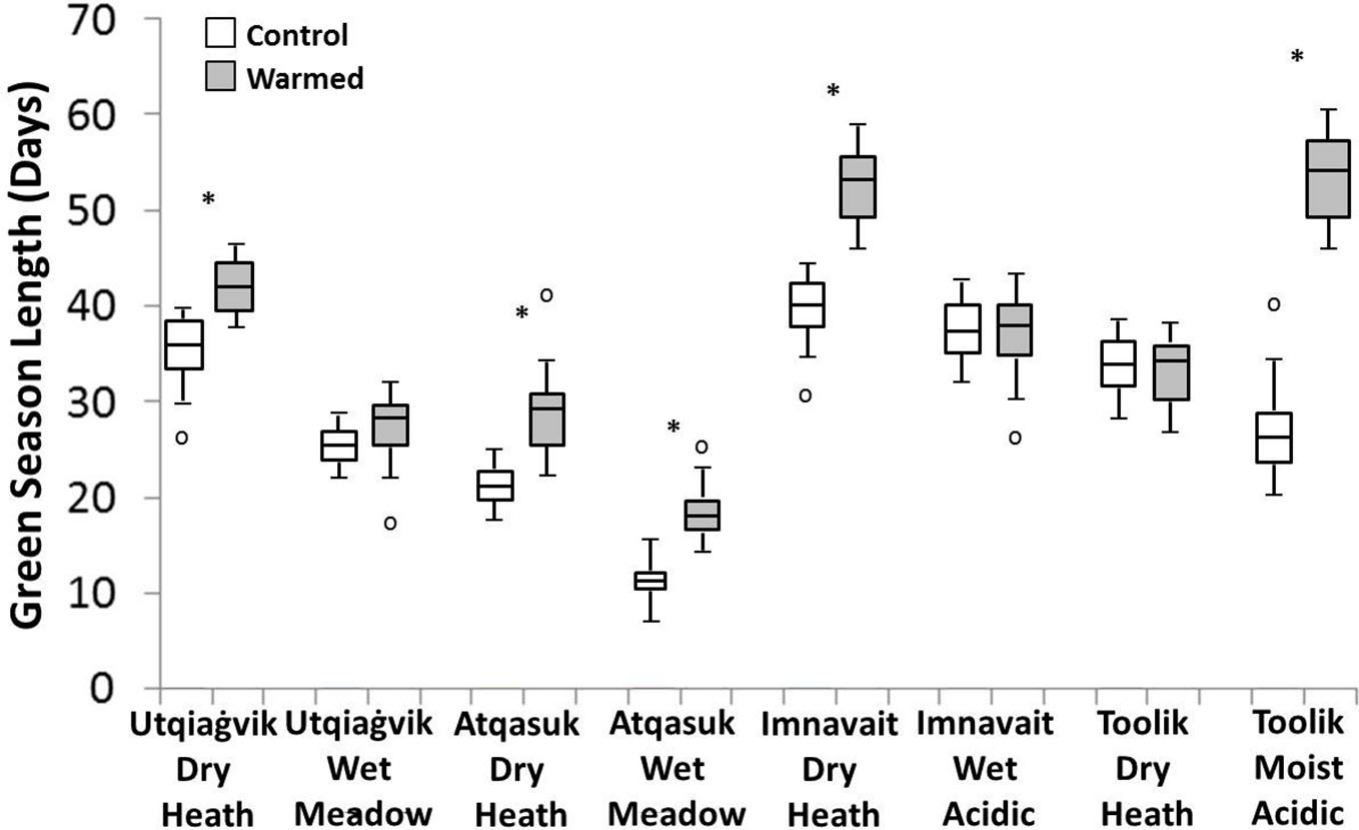
Green season length in control (open) and warmed (filled) plots at the eight study sites. A * denotes a statistically significant difference between treatments at the site (based on ANOVA, p < 0.05).

On average, OTC warming treatment increased the length of green season, although the magnitude of the effects varied among sites (**Figure 4**). Imnavait Creek dry and Toolik Lake moist sites showed the largest increases in green season length (+13 days p<0.001 and +28 days p<0.001 respectively), while the Imnavait Creek wet and Toolik Lake dry sites showed no change (+1 day p=0.613 and +0 day p=0.822 respectively). Both the Atqasuk dry (+8 days p<0.001) and wet (+7 days p<0.001) sites increased in green season length with OTC warming. The effects of warming on green season length was mixed at the Utqiaġvik sites with the dry increasing in length (+6 days p=0.008) and the wet remained unchanged (+3 days p=0.211).

### NDVI and Cover of Plant Growth Forms

In general, bryophytes and graminoids were associated with a delayed peak in NDVI and shrubs and lichens were associated with an earlier peak in NDVI (**Table 3**). The cover of bryophytes was positively correlated with the timing of peak NDVI at two sites (Utqiaġvik dry r= 0.39 p=0.032 and Atqasuk dry r=0.36 p=0.047), while lichen cover was negatively correlated with the timing of peak NDVI at the Imnavait Creek dry site (r=-0.36 p=0.048). Forbs were negatively correlated with the timing of peak NDVI at the Utqiaġvik wet site (r=-0.51 p=0.010). Graminoids were positively correlated with the timing of peak NDVI at both the Atqasuk (r=0.54 p=0.005) and Imnavait Creek (r=0.45 p=0.026) wet sites. Shrubs were negatively correlated with the timing of peak NDVI; the relationship was significant at two sites for deciduous shrubs (Imnavait Creek dry r=-0.42, p=0.037 and Toolik Lake moist r=-0.54, p=0.006) and at four sites for evergreen shrubs (Utqiaġvik dry r=-0.46, p=0.023, Imnavait Creek dry r=-0.44, p0.035, Imnavait Creek wet r=-0.43, p=0.037, and Toolik Lake dry r=-0.48, p=0.026).

**Table 3:** Pearson correlations (r) between the cover of each plant growth form and the magnitude (Value) or timing (day of the year; DOY) of peak NDVI at the eight study sites.

Site	Bryophyte				Lichen				Forb				Graminoid				Deciduous Shrub				Evergreen Shrub			
			Peak NDVI				Peak NDVI				Peak NDVI				Peak NDVI				Peak NDVI				Peak NDVI		
	Cover	(SD)	Value	DOY	Cover	(SD)	Value	DOY	Cover	(SD)	Value	DOY	Cover	(SD)	Value	DOY	Cover	(SD)	Value	DOY	Cover	(SD)	Value	DOY
Utqiaġvik																								
Dry	5.0	(5.3)	0.02	**0.39**	11.9	(9.8)	-0.16	0.35	3.8	(3.7)	-0.09	0.11	22.2	(13.7)	-0.14	0.08	8.8	(3.9)	**0.58**	-0.17	19.8	(10.5)	**0.42**	**-0.46**
Wet	3.1	(3.0)	0.20	0.22	1.8	(1.0)	-0.02	-0.20	4.0	(3.5)	-0.26	**-0.51**	45.6	(12.5)	**0.54**	0.24	1.6	(4.2)	0.19	0.22	0.0	(0)	–	–
Atqasuk																								
Dry	4.0	(3.7)	-0.01	0.08	14.2	(5.8)	**-0.44**	0.23	1.2	(1.9)	**-0.50**	-0.36	14.2	(12.2)	**0.41**	0.08	1.3	(2.3)	0.20	0.20	20.9	(7.4)	0.26	-0.29
Wet	3.9	(3.1)	-0.17	**0.36**	0.0	(0)	–	–	0.0	(0.2)	0.01	-0.17	58.1	(7.1)	**0.43**	**0.54**	4.0	(3.2)	**0.60**	0.20	0.0	(0)	–	–
Imnavait Creek																								
Dry	4.0	(3.3)	0.15	0.27	12.1	(9.6)	-0.35	**-0.36**	2.2	(1.8)	0.13	0.10	17.8	(11.2)	0.24	-0.02	9.0	(6.5)	**0.37**	**-0.42**	25.1	(12.1)	**0.51**	**-0.44**
Wet	5.6	(4.3)	0.13	0.28	2.2	(1.9)	-0.04	-0.13	2.3	(0.8)	0.14	0.33	41.2	(15.6)	**0.44**	**0.45**	11.6	(7.4)	0.30	-0.31	6.7	(4.2)	**0.46**	**-0.43**
Toolik Lake																								
Dry	2.3	(1.9)	0.11	0.14	26.3	(8.3)	-0.24	-0.26	9.2	(5.4)	**0.36**	0.13	7.6	(5.8)	0.20	-0.01	12.1	(8.1)	0.24	-0.25	17.2	(10.1)	**0.52**	**-0.48**
Moist	6.7	(4.1)	0.24	0.18	1.2	(0.9)	-0.02	-0.11	0.9	(0.6)	0.20	0.19	14.0	(9.7)	**0.39**	0.26	42.0	(18.1)	**0.38**	**-0.54**	8.7	(4.9)	**0.38**	-0.24

The average plot cover (Cover; measured at the top of the canopy) and the standard deviation (SD) among plots are provided. Bold indicates a statistically significant Pearson correlation value (p < 0.05).

The relationship between plant growth form cover and magnitude of peak NDVI also varied by site, with graminoids and shrubs showing the strongest correlations (**Table 3**). Bryophytes were not correlated with the magnitude of peak NDVI at any of the study sites. Lichens were negatively correlated with the magnitude of peak NDVI at the Atqasuk dry site (r=-0.44 p=0.032). Forbs were negatively correlated with the magnitude of peak NDVI at the Atqasuk dry site (r=-0.50 p=0.013) and positively correlated at the Toolik Lake dry site (r=0.36 p=0.048). Graminoids were positively correlated with the magnitude of peak NDVI at five of the study sites (Utqiaġvik dry r=0.54 p=0.006, Atqasuk dry r=0.41 p=0.038, Atqasuk wet r=0.43 p=0.029, Imnavait Creek wet r=0.44 p=0.34, and Toolik Lake moist r=0.39 p=0.036). Deciduous shrubs were positively correlated with the magnitude of peak NDVI at four of the study sites (Utqiaġvik dry r=0.58 p=0.002, Atqasuk wet r=0.60 p=0.002, Imnavait Creek dry r=0.37 p=0.039, and Toolik Lake moist r=0.38 p=0.037). Evergreen shrubs were positively correlated with the magnitude of peak NDVI at five of the study sites (Utqiaġvik dry r=0.42 p=0.040, Imnavait Creek dry r=0.51 p=0.012, Imnavait Creek wet r=0.46 p=0.028, Toolik Lake dry r=0.52 p=0.008, and Toolik Lake moist r=0.38 p=0.045).

## Discussion

As anticipated, experimental warming increased canopy surface temperatures across all eight study sites. However, the magnitude of the warming effect was season and site dependent, with not all sites and months warming significantly. Full-season warming values from OTCs ranged between +0.47 to +3.14°C, which were similar to those previously reported (Hollister et al., 2006; Bokhorst et al., 2013). Contributing factors to variation in experimental warming among sites include latitude, sky conditions, sun angle, and wind conditions, with the southernmost sites demonstrating the most pronounced warming (Marion et al., 1997; Bokhorst et al., 2013). Higher sun angle and resulting increased solar radiation may also explain the increased warming effect during June and July at most sites. Late in the growing season, specifically after the first sunset, OTC effects decrease as the daily incoming solar radiation begins to decrease.

Near-daily measures of plot level NDVI showed experimental warming increased peak NDVI at most sites, but as might be expected given the differences in the effectiveness of OTC warming, the warming response of peak NDVI varied by community type and across the latitudinal gradient. The southernmost study sites demonstrated the largest increases in peak NDVI despite the difference in duration of warming treatment between Toolik Lake (23 seasons) and Imnavait Creek (two seasons). However, the differences in the NDVI response may be a result of effects of size of the temperature increase. The enhanced NDVI values were present in all Toolik Lake and Imnavait Creek plots, suggesting that species differences (e.g. *Eriophorum vaginatum* or *Arctostaphylos alpina* dominance), and the ability of these species to respond to warming may be driving factors in NDVI increases (Boelman et al., 2003; Walker et al., 2003). Moist or wet sites were more responsive to warming than their complimentary dry site from the same region. Differences in warming responses among these community types may be a result of community attributes, such as differences in productivity (La Puma et al., 2007), aboveground biomass (Boelman et al., 2003; Hudson and Henry, 2009; Hollister et al., 2015), and duration of warming treatments. Warmer temperatures may also contribute to increased soil moisture loss which is more likely to be important in dry communities. Studies in alpine regions have reported delays in phenological events and shortened growing season in response to warming-induced drying (Jonas et al., 2008; Dorji et al., 2013).

Peak NDVI was generally positively correlated with graminoid and shrub (both deciduous and evergreen) cover. The influence of graminoids and shrubs were greater in wet or moist communities due to the inherently high graminoid and shrub cover in these ecosystems compared to the more sparsely vegetated dry tundra (Welker et al., 1997; Welker et al., 1999; Hollister et al., 2015). The positive influence of graminoids and shrubs (i.e. relatively taller-statured plants) was in contrast to the marginal effects of lichen and bryophyte growth forms. Lichens and bryophytes may have their signal marginalized by the color differences inherent in each of the species. This disparity of growth form influence on peak NDVI supports the findings of previous studies that found positive relationships between vegetation biomass and NDVI values (Boelman et al., 2003; Riedel et al., 2005; Raynolds et al., 2008; Berner et al., 2018). Increased graminoid and deciduous shrub cover also generally shifted the timing of peak NDVI to later in the growing season, as these growth forms leaf out later and continue to increase aboveground biomass as the season progresses. Evergreen shrubs were associated with earlier peak NDVI as well, and this result reflects their leaf longevity (Karlsson, 1992).

Accelerated spring green-up and delayed senescence of tundra resulting in increased season length in response to warming was a postulated outcome of climate change proposed in a conceptual model early in the ITEX program (Welker et al., 1997) and are consistent with previous findings (Arft et al., 1999; Aerts et al., 2006; May et al., 2017). Increases in spring phenological progression and delayed senescence as result of increased temperature have been well documented and could lengthen the green season in the Arctic (Wookey et al., 1993; Oberbauer et al., 1998; Arft et al., 1999; Starr et al., 2000; Marchand et al., 2004; Oberbauer et al., 2013; Khorsand Rosa et al., 2015; Prevéy et al., 2017). The ability of individual species to take advantage of warming temperatures and other resulting environmental conditions (e.g. precipitation rates, soil moisture, more snow) may be a major component driving documented community composition shifts (Arft et al., 1999; Walker et al., 2006; Hollister et al., 2015) and ecosystem function changes (Oechel et al., 1992; Boelman et al., 2003; Schimel and Bennett, 2004; Welker et al., 2005; La Puma et al., 2007; Leffler et al., 2016; Jespersen et al., 2018). Lengthening of the green season will likely favor some species over others and ultimately result in changes in phenology, species composition and possibly trace gas feedbacks (Bjorkman et al., 2015; Khorsand Rosa et al., 2015; Kelsey et al., 2016). These changes are likely to impact the timing and magnitude of forage availability which will have cascading implications on food-web dynamics (Welker et al., 2005; Post et al., 2009; Myers-Smith et al., 2011; Richert et al., 2019).

The responsiveness of timing of peak NDVI to warming differed by site and species composition. Peak NDVI occurred earlier in response to warming in wet and moist communities, dominated by graminoids and deciduous shrubs, with the greatest shifts in the southernmost sites. This finding suggests that variation in the species that constitute different growth forms may cause communities to respond at different rates to warming. Variations in community dominance and ground cover have been previously shown to alter community NDVI values (Raynolds et al., 2008; Pattison et al., 2015). The Atqasuk and Toolik Lake dry sites are dominated by lichens and are more sparsely vegetated, and both sites showed peak NDVI was delayed with warming. Alternatively, the Utqiaġvik and Imnaviat Creek dry sites have a greater cover of evergreen shrubs and these sites showed peak NDVI was earlier with warming. Therefore, it is reasonable to conclude that the dominant vegetation growth form and cover extent have an effect on the responsiveness of a community type to warming, and any dominance shifts will likely impact NDVI directly and also influence how responsive NDVI is to changing temperatures in the future.

The variability of the magnitude and timing of peak NDVI across our study region shows that similar communities do not respond uniformly across a region. This response pattern begins to shed some light on why some regions green more quickly than others (Jia et al., 2003; Stow et al., 2007; Bhatt et al., 2013). Differences in the most southerly sites (Toolik Lake and Imnavait Creek) may also be a result of differences in warming treatment duration, where Toolik Lake has likely had ample time for community composition changes while Imnavait Creek vegetation is responding to novel warming. Our findings also highlight the value of coupling traditional visually-assessed Arctic plant community changes with small-scale remotely-sensed observations. The synthesis of these two methodologies will allow future studies to thoroughly investigate community-scale changes and their impact on broader landscape-scale patterns, with insight into mechanisms that drive change such as browning in regions of the Arctic (Verbyla, 2008; Phoenix and Bjerke, 2016). Understanding variations in community change mechanisms across latitudinal gradients is critical for the ability to accurately scale up landscape cover change predictions. It should be noted that the shorter duration of warming at Imnavait Creek (2 vs. ≥ 20 years) may have played a role in the magnitude of NDVI differences between treatments. Community changes associated with warming would likely impact NDVI values through the differences in growth form cover that we report here and by previous studies (Arft et al., 1999; Elmendorf et al., 2012a, Elmendorf et al., 2012b, Bjorkman et al., 2015).

Our findings also highlight the value of incorporation of ground-based remotely-sensed measurements at sites across the Arctic where detailed vegetation dynamics are also monitored. This study is the first that investigates the effects of warming on daily NDVI changes and demonstrates the complexity of scaling across heterogeneous tundra communities. Our results suggest the non-uniformity of warming effects on the magnitude and timing of peak NDVI across northern Alaska. The fact that peak NDVI was not markedly increased at most sites may be misleading given that spring greening was hastened and green season lengthened. These changes suggest that while peak NDVI may not increase markedly, the lengthening of the green season in response to warming is likely to result in greater productivity. Collectively, it appears that in a warmer Arctic (as simulated in our experiment) there may be a host of organismal and ecosystem process changes, including the period of carbon gain and greater net primary productivity as well as shifts in the timing of vegetation phenology that may have consequences for foraging ecology of ungulates and other trophic interactions.

## Data Availability Statement

The datasets generated for this study are available on request to the corresponding author.

## Author Contributions

JM led data collection and analysis and co-wrote manuscript with RH and KB. RH and KB co-wrote manuscript and revisions. KB and JH assisted in data collection on the project. RH, CT, JW, WG, and SO contributed data for this manuscript, advised in revisions, and secured funding for the project.

## Funding

This study was conducted with support from National Science Foundation grants PLR-1504381 and PLR-1836898.

## Conflict of Interest

The authors declare that the research was conducted in the absence of any commercial or financial relationships that could be construed as a potential conflict of interest.

The handling Editor declared a past co-authorship with three of the authors [RH], [SO], and [JW].

## References

[B1] ACIA (2005). Arctic Climate Impact Assessment 2004 (Cambridge, UK: Cambridge University Press).

[B2] Aerts R. Cornelissen J. H. C. Dorrepaal E. (2006). Plant performance in a warmer world: General responses of plants from cold, northern biomes and the importance of winter and spring events. Plant Ecol. 182 (1–2), 65–77. 10.1007/978-1-4020-4443-4_5

[B3] Arft A. M. Walker M. D. Gurevitch J. E. T. A. Alatalo J. M. Bret-Harte M. S. Dale M. (1999). Responses of tundra plants to experimental warming: meta-analysis of the international tundra experiment. Ecol. Mono. 69 (4), 491–511. 10.2307/2657227

[B4] Barrett R. T. Hollister R. D. Oberbauer S. F. Tweedie C. E. (2015). Arctic plant responses to changing abiotic factors in northern Alaska. Amer. J. Bot. 102 (12), 2020–2031. 10.3732/ajb.1400535 26672012

[B5] Berner L. T. Jantz P. Tape K. D. Goetz S. J. (2018). Tundra plant above-ground biomass and shrub dominance mapped across the North Slope of Alaska. Env. Res. Lett. 13 (3), 035002. 10.1088/1748-9326/aaaa9a

[B6] Bhatt U. S. Walker D. A. Raynolds M. K. Bieniek P. A. Epstein H. E. Comiso J. C. (2013). Recent declines in warming and vegetation greening trends over pan-Arctic tundra. Rem. Sens. 5 (9), 4229–4254. 10.3390/rs5094229

[B7] Billings W. D. Bliss L. C. (1959). An alpine snowbank environment and its effects on vegetation, plant development, and productivity. Ecol 40 (3), 388–397. 10.2307/1929755

[B8] Bjorkman A. D. Elmendorf S. C. Beamish A. L. Vellend M. Henry G. H. (2015). Contrasting effects of warming and increased snowfall on Arctic tundra plant phenology over the past two decades. Glob. chng. bio. 21 (12), 4651–4661. 10.1111/gcb.13051 26216538

[B9] Bjorkman A. D. Myers-Smith I. H. Elmendorf S. C. Normand S. Rüger N. Beck P. S. (2018). Plant functional trait change across a warming tundra biome. Nat 562 (7725), 57–62. 10.1038/s41586-018-0563-7 30258229

[B10] Blok D. Schaepman-Strub G. Bartholomeus H. Heijmans M. M. Maximov T. C. Berendse F. (2011). The response of Arctic vegetation to the summer climate: relation between shrub cover, NDVI, surface albedo and temperature. Env. Res. Lett. 6 (3), 035502. 10.1088/1748-9326/6/3/035502

[B11] Boelman N. T. Stieglitz M. Rueth H. M. Sommerkorn M. Griffin K. L. Shaver G. R. (2003). Response of NDVI, biomass, and ecosystem gas exchange to long term warming and fertilization in wet sedge tundra. Oecol 135 (3), 414–421. 10.1007/s00442-003-1198-3 12721832

[B12] Boelman N. T. Stieglitz M. Griffin K. L. Shaver G. R. (2005). Inter-annual variability of NDVI in response to long-term warming and fertilization in wet sedge and tussock tundra. Oeco 143 (4), pp.588–pp.597. 10.1007/s00442-005-0012-9 15812655

[B13] Bokhorst S. Huiskes A. D. Aerts R. Convey P. Cooper E. J. Dalen L. (2013). Variable temperature effects of Open Top Chambers at polar and alpine sites explained by irradiance and snow depth. Glob. chng. boil. 19 (1), 64–74. 10.1111/gcb.12028 23504721

[B14] Callaghan T. V. Press M. C. Lee J. A. Robinson D. L. Anderson C. W. (1999). Spatial and temporal variability in the responses of Arctic terrestrial ecosystems to environmental change. Pol. Res. 18 (2), 191–197. 10.1111/j.1751-8369.1999.tb00293.x

[B15] Callaghan T. V. Johansson M. Brown R. D. Groisman P. Y. Labba N. Radionov V. (2011). The changing face of Arctic snow cover: A synthesis of observed and projected changes. Ambio 40 (1), 17–31. 10.1007/s13280-011-0212-y

[B16] Chapin F. S. III Shaver G. R. (1985). Individualistic growth response of tundra plant species to environmental manipulations in the field. Ecol 66 (2), 564–576. 10.2307/1940405

[B17] Dorji T. Totland Ø. Moe S. R. Hopping K. A. Pan J. Klein J. A. (2013). Plant functional traits mediate reproductive phenology and success in response to experimental warming and snow addition in Tibet. Glob. chng. bio. 19 (2), 459–472. 10.1111/gcb.12059 23504784

[B18] Elmendorf S. C. Henry G. H. Hollister R. D. Björk R. G. Boulanger-Lapointe N. Cooper E. J. (2012a). Plot-scale evidence of tundra vegetation change and links to recent summer warming. Nat. Clim. Chng. 2 (6), 453–457. 10.1038/nclimate1465

[B19] Elmendorf S. C. Henry G. H. Hollister R. D. Björk R. G. Bjorkman A. D. Callaghan T. V. (2012b). Global assessment of experimental climate warming on tundra vegetation: heterogeneity over space and time. Ecol. let. 15 (2), 164–175. 10.1111/j.1461-0248.2011.01716.x 22136670

[B20] Elmendorf S. C. Henry G. H. Hollister R. D. Fosaa A. M. Gould W. A. Hermanutz L. (2015). Experiment, monitoring, and gradient methods used to infer climate change effects on plant communities yield consistent patterns. Proc. Nat. Acad. Sci. 112 (2), 448–452. 10.1073/pnas.1410088112 25548195 PMC4299205

[B21] Evans B. M. Walker D. A. Benson C. S. Nordstrand E. A. Petersen G. W. (1989). Spatial interrelationships between terrain, snow distribution and vegetation patterns at an arctic foothills site in Alaska. Ecog 12 (3), 270–278. 10.1111/j.1600-0587.1989.tb00846.x

[B22] Gamon J. A. Huemmrich K. F. Stone R. S. Tweedie C. E. (2013). Spatial and temporal variation in primary productivity (NDVI) of coastal Alaskan tundra: Decreased vegetation growth following earlier snowmelt. Rem. Sens. Env. 129, 144–153. 10.1016/j.rse.2012.10.030

[B23] Guay K. C. Beck P. S. Berner L. T. Goetz S. J. Baccini A. Buermann W. (2014). Vegetation productivity patterns at high northern latitudes: a multi-sensor satellite data assessment. Glob. Chng. Bio. 20 (10), 3147–3158. 10.1111/gcb.12647 PMC4312854 24890614

[B24] Healey N. C. Oberbauer S. F. Ahrends H. E. Dierick D. Welker J. M. Leffler A. J. (2014). A Mobile Instrumented Sensor Platform for LongTerm Terrestrial Ecosystem Analysis: An Example Application in an Arctic Tundra Ecosystem. J. Env. Info. 24 (1), 1–10. 10.3808/jei.201400278

[B25] Henry G. H. R. Molau U. (1997). Tundra plants and climate change: the International Tundra Experiment (ITEX). Glob. Chng. Bio. 3 (S1), 1–9. 10.1111/j.1365-2486.1997.gcb132.x

[B26] Hobbie S. E. Finlay J. C. Janke B. D. Nidzgorski D. A. Millet D. B. Baker L. A. (2017). Contrasting nitrogen and phosphorus budgets in urban watersheds and implications for managing urban water pollution. Proc. Nat. Acad. Sci. 114 (16), 4177–4182. 10.1073/pnas.1618536114 28373560 PMC5402417

[B27] Hollister R. D. Webber P. J. Bay C. (2005). Plant response to temperature in northern Alaska: implications for predicting vegetation change. Ecol 86 (6), 1562–1570. 10.1890/04-0520

[B28] Hollister R. D. Webber P. J. Nelson F. E. Tweedie C. E. (2006). Soil thaw and temperature response to air warming varies by plant community: Results from an open-top chamber experiment in northern Alaska. Arc. Ant. Alp. Res. 38, 206–215. 10.1657/1523-0430(2006)38[206:STATRT]2.0.CO;2

[B29] Hollister R. D. May J. L. Kremers K. S. Tweedie C. E. Oberbauer S. F. Liebig J. A. (2015). Warming experiments elucidate the drivers of observed directional changes in tundra vegetation. Ecol. Evol. 5 (9), 1881–1895. 10.1002/ece3.1499 26140204 PMC4485969

[B30] Hudson J. M. Henry G. H. (2009). Increased plant biomass in a High Arctic heath community from 1981 to 2008. Ecol 90 (10), 2657–2663. 10.1890/09-0102.1 19886474

[B31] Huemmrich K. F. Gamon J. A. Tweedie C. E. Campbell P. K. E. Landis D. R. Middleton E. M. (2013). Arctic tundra vegetation functional types based on photosynthetic physiology and optical properties. IEEE J. Sel. Top. Appl. Earth Obs. Remote Sens. 6 (2), 265–275. 10.1109/JSTARS.2013.2253446

[B32] Jespersen R. G. Leffler A. J. Oberbauer S. F. Welker J. M. (2018). Arctic plant ecophysiology and water source utilization in response to altered snow: isotopic (δ 18 O and δ 2 H) evidence for meltwater subsidies to deciduous shrubs. Oecol 187 (4), 1009–1023. 10.1007/s00442-018-4196-1 29955988

[B33] Jia G. J. Epstein H. E. Walker D. A. (2003). Greening of Arctic Alaska 1981-2001. Geophys. Res. Lett. 30 (20), 2067. 10.1029/2003GL018268

[B34] Jonas T. Rixen C. Sturm M. Stoeckli V. (2008). How alpine plant growth is linked to snow cover and climate variability. J. Geophys. Res.: Biogeosci. 113 (G3). 10.1029/2007JG000680

[B35] Ju J. Masek J. G. (2016). The vegetation greenness trend in Canada and US Alaska from 1984-2012 Landsat data. Rem. Sens. Env. 176, 1–16. 10.1016/j.rse.2016.01.001

[B36] Karlsson P. S. (1992). Leaf longevity in evergreen shrubs: variation within and among European species. Oecol 91 (3), 346–349. 10.1007/BF00317622 28313541

[B37] Keenan T. F. Riley K. J. (2018). Greening of the land surface in the world’s cold regions consistent with recent warming. Nat. Clim. Chng. 8 (9), 825. 10.1038/s41558-018-0258-y PMC6180328 30319714

[B38] Kelsey K. C. Leffler A. J. Beard K. H. Schmutz J. A. Choi R. T. Welker J. M. (2016). Interactions among vegetation, climate, and herbivory control greenhouse gas fluxes in a subarctic coastal wetland. J. Geophys. Res.: Biogeosci. 121 (12), 2960–2975. 10.1002/2016JG003546

[B39] Kerr J. T. Ostrovsky M. (2003). From space to species: ecological applications for remote sensing. Trend. Ecol. Evol. 18 (6), 299–305. 10.1016/S0169-5347(03)00071-5

[B40] Khorsand Rosa R. Oberbauer S. F. Starr G. Parker La Puma I. Pop E. Ahlquist L. (2015). Plant phenological responses to a long-term experimental extension of growing season and soil warming in the tussock tundra of Alaska. Glob. Chng. Bio. 21 (12), 4520–4532. 10.1111/gcb.13040 26183112

[B41] Kriegler F. J. Malila M. A. Nalepka R. F. Richardson W. (1969). Preprocessing transformations and their effects on multispectral recognition. Proceedings of the Sixth International Symposium on Remote Sensing of Environment (Ann Arbor, MI: University of Michigan), 97–131.

[B42] La Puma I. P. Philippi T. E. Oberbauer S. F. (2007). Relating NDVI to ecosystem CO2 exchange patterns in response to season length and soil warming manipulations in arctic Alaska. Rem. Sens. Env. 109 (2), 225–236. 10.1016/j.rse.2007.01.001

[B43] Lara M. J. Nitze I. Grosse G. Martin P. McGuire A. D. (2018). Reduced arctic tundra productivity linked with landform and climate change interactions. Sci. Rep. 8 (1), 2345. 10.1038/s41598-018-20692-8 29402988 PMC5799341

[B44] Leffler A. J. Klein E. S. Oberbauer S. F. Welker J. M. (2016). Coupled long-term summer warming and deeper snow alters species composition and stimulates gross primary productivity in tussock tundra. Oecol 181 (1), 287–297. 10.1007/s00442-015-3543-8 26747269

[B45] Liston G. E. Hiemstra C. A. (2011). The changing cryosphere: Pan-Arctic snow trends, (1979 2009). J. Clim. 24 (21), 5691–5712. 10.1175/JCLI-D-11-00081.1

[B46] Marchand F. L. Nijs I. Heuer M. Mertens S. Kockelbergh F. Pontailler J. Y. (2004). Climate warming postpones senescence in High Arctic tundra. Arc Ant. Alp. Res. 36 (4), 390–394. 10.1657/1523-0430(2004)036[0390:CWPSIH]2.0.CO;2

[B47] Marion G. M. Henry G. H. R. Freckman D. W. Johnstone J. Jones G. Jones M. H. (1997). Open-top designs for manipulating field temperature in high-latitude ecosystems. Glob. Chng. Bio. 3 (S1), 32. 10.1111/j.1365-2486.1997.gcb136.x

[B48] May J. Healey N. Ahrends H. Hollister R. Tweedie C. Welker J. (2017). Short-Term Impacts of the Air Temperature on Greening and Senescence in Alaskan Arctic Plant Tundra Habitats. Rem. Sens. 9 (12), 1338. 10.3390/rs9121338

[B49] Molau U. Mölgaard P. (1996). International Tundra Experiment (ITEX) Manual (Copenhagen, Denmark: Danish Polar Center).

[B50] Myers-Smith I. H. Forbes B. C. Wilmking M. Hallinger M. Lantz T. Blok D. (2011). Shrub expansion in tundra ecosystems: dynamics, impacts and research priorities. Env. Res. Lett. 6 (4), 045509. 10.1088/1748-9326/6/4/045509

[B51] Myers-Smith I. H. Elmendorf S. C. Beck P. S. Wilmking M. Hallinger M. Blok D. (2015). Climate sensitivity of shrub growth across the tundra biome. Nat. Clim. Chng. 5 (9), 887–891. 10.1038/nclimate2697

[B52] Myers-Smith I. H. Kerby J. T. Phoenix G. K. Bjerke J. W. Epstein H. E. Assmann J. J. (2020). Complexity revealed in the greening of the Arctic. Nat. Clim. Chng. 10 (2), 106–117. 10.1038/s41558-019-0688-1

[B53] Oberbauer S. F. Starr G. Pop E. W. (1998). Effects of extended growing season and soil warming on carbon dioxide and methane exchange of tussock tundra in Alaska. J. Geophys. Res. 103, 29075–29082. 10.1029/98JD00522

[B54] Oberbauer S. F. Elmendorf S. C. Troxler T. G. Hollister R. D. Rocha A. V. Bret-Harte M. S. (2013). Phenological response of tundra plants to background climate variation tested using the International Tundra Experiment. Phil. Trans. R. Soc.: Biol. Sci. 368 (1624), 20120481. 10.1098/rstb.2012.0481 PMC3720054 23836787

[B55] Oechel W. C. Hastings S. J. Vourlitis G. Jenkins M. Riechers G. Grulke N. (1992). Recent change of Arctic tundra ecosystems from a net carbon dioxide sink to a source. Nat 361 (6412), 520–523. 10.1038/361520a0

[B56] Ostendorf B. Reynolds J. F. (1998). A model of arctic tundra vegetation derived from topographic gradients. Landsc. Ecol. 13 (3), 187–201. 10.1023/A:1007986410048

[B57] Parsons A. N. Welker J. M. Wookey P. A. Press M. A. Callaghan T. V. Lee J. A. (1994). Growth responses of four sub-Arctic dwarf shrubs to simulated environmental change. J. Ecol., 307–318. 10.2307/2261298

[B58] Pattison R. R. Jorgenson J. C. Raynolds M. K. Welker J. M. (2015). Trends in NDVI and tundra community composition in the Arctic of NE Alaska between 1984 and 2009. Ecosystems 18 (4), 707–719. 10.1007/s10021-015-9858-9

[B59] Pearson R. G. Phillips S. J. Loranty M. M. Beck P. S. Damoulas T. Knight S. J. (2013). Shifts in Arctic vegetation and associated feedbacks under climate change. Nat. Clim. Chng. 3 (7), 673–677. 10.1038/nclimate1858

[B60] Phoenix G. K. Bjerke J. W. (2016). Arctic browning: extreme events and trends reversing arctic greening. Glob. Chng. Bio. 22 (9), 2960–2962. 10.1111/gcb.13261 27095022

[B61] Post E. Forchhammer M. C. Bret-Harte M. S. Callaghan T. V. Christensen T. R. Elberling B. (2009). Ecological dynamics across the Arctic associated with recent climate change. Science 325 (5946), 1355–1358. 10.1126/science.1173113 19745143

[B62] Prevéy J. Vellend M. Rüger N. Hollister R. D. Bjorkman A. D. Myers-Smith I. H. (2017). Greater temperature sensitivity of plant phenology at colder sites: implications for convergence across northern latitudes. Glob. Chng. Bio. 23 (7), 2660–2671. 10.1111/gcb.13619 28079308

[B63] R Core Team (2018). R: A language and environment for statistical computing (Vienna, Austria.: R Foundation for Statistical Computing). URL https://www.R-project.org/.

[B64] Raynolds M. K. Comiso J. C. Walker D. A. Verbyla D. (2008). Relationship between satellite derived land surface temperatures, arctic vegetation types, and NDVI. Rem. Sens. Env. 112 (4), 1884–1894. 10.1016/j.rse.2007.09.008

[B65] Reichle L. M. Epstein H. E. Bhatt U. S. Raynolds M. K. Walker D. A. (2018). Spatial Heterogeneity of the Temporal Dynamics of Arctic Tundra Vegetation. Geophys. Res. Lett. 45 (17), 9206–9215. 10.1029/2018GL078820

[B66] Richert J. Leffler J. Spalinger D. Welker J. M. (2019). Snowier winters extend autumn availability of high-quality forage for caribou in Arctic Alaska. Alaska Sect. of Wildlife Soc., Oral presentation, January 2020. (The Wildlife Society)

[B67] Riedel S. M. Epstein H. E. Walker D. A. (2005). Biotic controls over spectral reflectance of arctic tundra vegetation. Int. J. Rem. Sens. 26 (11), 2391–2405. 10.1080/01431160512331337754

[B68] Shaefer J. A. Messier F. (1995). Scale-dependent correlations of arctic vegetation and snow cover. Arc. Alp. Res. 27 (1), 38–43. 10.2307/1552066

[B69] Schimel J. P. Bennett J. (2004). Nitrogen mineralization: challenges of a changing paradigm. Ecol. 85 (3), 591–602. 10.1890/03-8002

[B70] Serreze M. C. Barrett A. P. Stroeve J. C. Kindig D. M. Holland M. M. (2009). The emergence of surface-based Arctic amplification. Cryosph 3, 11–19. 10.5194/tc-3-11-2009

[B71] Starr G. Oberbauer S. F. Pop E. W. (2000). Effects of extended growing season and soil warming on phenology and physiology of *Polygonum bistorta.* Glob. Chng. Bio. 6, 357 369. 10.1046/j.1365-2486.2000.00316.x

[B72] Stocker T. F. Qin D. Plattner G. K. Tignor M. Allen S. K. Boschung J. (2013). Climate change 2013: The physical science basis. Contribution of working group I to the fifth assessment report of the intergovernmental panel on climate change. 1535.

[B73] Stow D. Petersen A. Hope A. Engstrom R. Coulter L. (2007). Greenness trends of Arctic tundra vegetation in the 1990s: comparison of two NDVI data sets from NOAA AVHRRsystems. Int. J. Rem. Sens. 28 (21), 4807–4822. 10.1080/01431160701264284

[B74] Tape K. Sturm M. Racine C. (2006). The evidence for shrub expansion in northern Alaska and the Pan-Arctic. Glob. Chng. Bio. 12 (4), 686–702. 10.1111/j.1365-2486.2006.01128.x

[B75] Tape K. D. Hallinger M. Welker J. M. Ruess R. W. (2012). Landscape heterogeneity of shrub expansion in Arctic Alaska. Ecosys 15 (5), 711–724. 10.1007/s10021-012-9540-4

[B76] Verbyla D. (2008). The greening and browning of Alaska based on 1982-2003 satellite data. Glob. Ecol. Biogeo. 17 (4), 547–555. 10.1111/j.1466-8238.2008.00396.x

[B77] Walker D. A. Binnian E. Evans B. M. Lederer N. D. Nordstrand E. Webber P. J. (1989). Terrain, vegetation and landscape evolution of the R4D research site, Brooks Range Foothills, Alaska. Ecogr 12 (3), 238–261. 10.1111/j.1600-0587.1989.tb00844.x

[B78] Walker D. A. Epstein H. E. Jia G. J. Balser A. Copass C. Edwards E. J. (2003). Phytomass, LAI, and NDVI in northern Alaska: Relationships to summer warmth, soil pH, plant functional types, and extrapolation to the circumpolar Arctic. J. Geophys. Res.: Atmos. 108 (D2). 10.1029/2001JD000986

[B79] Walker M. D. Wahren C. H. Hollister R. D. Henry G. H. Ahlquist L. E. Alatalo J. M. (2006). Plant community responses to experimental warming across the tundra biome. Proc. Nat. Acad. Of Sci. 103 (5), 1342–1346. 10.1073/pnas.0503198103 16428292 PMC1360515

[B80] Walker D. A. Epstein H. E. Raynolds M. K. Kuss P. Kopecky M. A. Frost G. V. (2012). Environment, vegetation and greenness (NDVI) along the North America and Eurasia Arctic transects. Env. Res. Lett. 7 (1), 015504. 10.1088/1748-9326/7/1/015504

[B81] Walker L. J. (1996). “Community baseline measurements for ITEX studies,” in International Tundra Experiment (ITEX) Manual. Eds. Molau U. Mölgaard P. (Copenhagen, Denmark: Danish Polar Center), 39–41.

[B82] Webber P. J. Walker M. D. (1991). International Tundra Experiment (ITEX):Resolution. Arc. Alp. Res. 23, 124.

[B83] Welker J. M. Molau U. Parsons A. N. Robinson C. H. Wookey P. A. (1997). Responses of *Dryas octopetala* to ITEX environmental manipulations: a synthesis with circumpolar comparisons. Glob. Chng. Bio. 3 (S1), 61–73. 10.1111/j.1365-2486.1997.gcb143.x

[B84] Welker J. M. Brown K. B. Fahnestock J. T. (1999). CO2 flux in arctic and alpine dry tundra: comparative field responses under ambient and experimentally warmed conditions. Arc. Ant. Alp. Res. 31 (3), 272–277. 10.1080/15230430.1999.12003309

[B85] Welker J. M. Fahnestock J. T. Sullivan P. F. Chimner R. A. (2005). Leaf mineral nutrition of Arctic plants in response to warming and deeper snow in northern Alaska. Oikos 109 (1), 167–177. 10.1111/j.0030-1299.2005.13264.x

[B86] Wookey P. A. Parsons A. N. Welker J. M. Potter J. A. Callaghan T. V. Lee J. A. (1993). Comparative responses of phenology and reproductive development to simulated environmental change in sub-arctic and high arctic plants. Oikos 490–502. 10.2307/3545361

[B87] Zesati S. A. V. (2017). Advancing High Spatial and Spectral Resolution Remote Sensing for Observing Plant Community Response to Environmental Variability and Change in the Alaskan Arctic (The University of Texas at El Paso).

[B88] Zhang Y. Song C. Band L. E. Sun G. Li J. (2017). Reanalysis of global terrestrial vegetation trends from MODIS products: Browning or greening? Rem. Sens. env. 191, 145–155. 10.1016/j.rse.2016.12.018

